# Can Limitations of Visuospatial Attention Be Circumvented? A Review

**DOI:** 10.3389/fpsyg.2017.01896

**Published:** 2017-10-27

**Authors:** Basil Wahn, Peter König

**Affiliations:** ^1^Institute of Cognitive Science, Universität Osnabrück, Osnabrück, Germany; ^2^Institut für Neurophysiologie und Pathophysiologie, Universitätsklinikum Hamburg-Eppendorf, Hamburg, Germany

**Keywords:** multisensory processing, visuospatial attention, joint action, attentional resources, multiple object tracking

## Abstract

In daily life, humans are bombarded with visual input. Yet, their attentional capacities for processing this input are severely limited. Several studies have investigated factors that influence these attentional limitations and have identified methods to circumvent them. Here, we provide a review of these findings. We first review studies that have demonstrated limitations of visuospatial attention and investigated physiological correlates of these limitations. We then review studies in multisensory research that have explored whether limitations in visuospatial attention can be circumvented by distributing information processing across several sensory modalities. Finally, we discuss research from the field of joint action that has investigated how limitations of visuospatial attention can be circumvented by distributing task demands across people and providing them with multisensory input. We conclude that limitations of visuospatial attention can be circumvented by distributing attentional processing across sensory modalities when tasks involve spatial as well as object-based attentional processing. However, if only spatial attentional processing is required, limitations of visuospatial attention cannot be circumvented by distributing attentional processing. These findings from multisensory research are applicable to visuospatial tasks that are performed jointly by two individuals. That is, in a joint visuospatial task requiring object-based as well as spatial attentional processing, joint performance is facilitated when task demands are distributed across sensory modalities. Future research could further investigate how applying findings from multisensory research to joint action research may facilitate joint performance. Generally, findings are applicable to real-world scenarios such as aviation or car-driving to circumvent limitations of visuospatial attention.

## 1. Introduction

In everyday life, humans continuously process information from several sensory modalities. However, the amount of information humans can process is limited (Marois and Ivanoff, 2005; Dux et al., 2006). In particular, using attentional mechanisms humans are able to selectively attend only a limited amount of information while neglecting irrelevant sensory input (James, 1890; Chun et al., 2011). Researchers have explained these limitations in terms of a limited pool of attentional resources that can be depleted under high attentional demands (Kahneman, 1973; Wickens, 2002; Lavie, 2005). These limitations do not solely apply to sensory processing but also to motor processing (e.g., see Pashler, 1994; Dux et al., 2006; Sigman and Dehaene, 2008), yet for this review we primarily focus on limitations in sensory processing.

Regarding the type of attentional demands, a distinction in attention research is that between object-based attention and spatial attention (Fink et al., 1997; Serences et al., 2004; Soto and Blanco, 2004). Object-based attention refers to selectively attending to features of an object (e.g., attending to the color or shape of an object) whereas spatial attention refers to selectively attending to a location in space.

In the present review, we will primarily focus on limitations of spatial attention in the visual sensory modality and on how they can be circumvented. We first review findings about these limitations with a focus on visuospatial tasks. We then briefly describe physiological correlates of attentional processing during visuospatial task performance. We then turn to review multisensory research that has investigated whether limitations in visuospatial attention can be circumvented by distributing information processing across several sensory modalities. Subsequently, we review research in which findings from multisensory research are applied to joint tasks (i.e., tasks that are performed jointly by two individuals). Finally, we conclude the review with future directions for research on how findings from multisensory research could be used to circumvent limitations of visuospatial attention in joint tasks.

## 2. Limitations of visuospatial attention and physiological correlates

**Figure 1:**
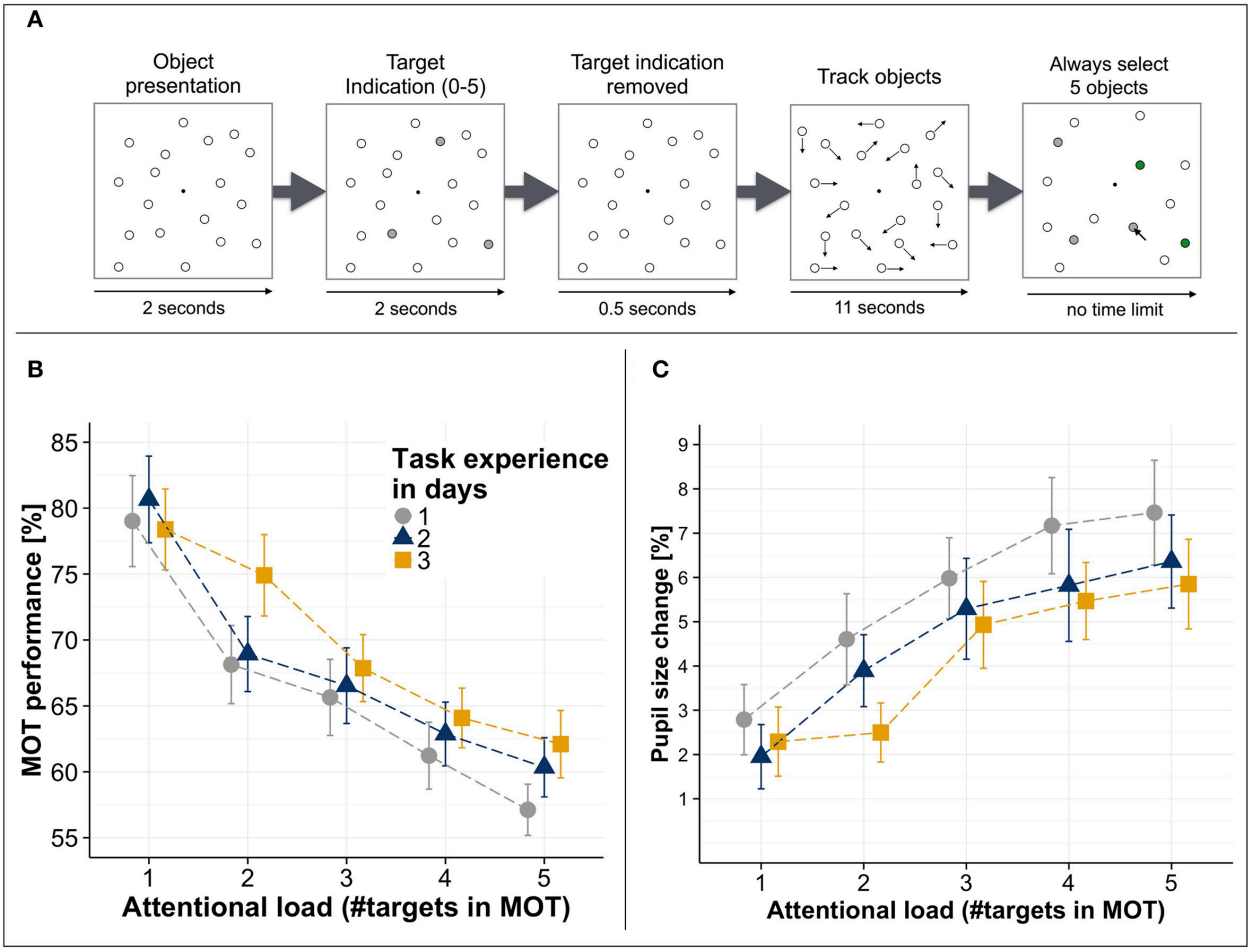
**(A)** Multiple object tracking (MOT) task trial logic. First, several stationary objects are shown on a computer screen. A subset of these objects is indicated as targets (here in gray). Then, the target indication is removed (i.e., targets become indistinguishable from the other objects) and all objects start moving randomly across the screen. After several seconds, the objects stop moving and participants are asked to select the previously indicated target objects. **(B)** MOT performance (i.e., percent correct of selected targets) as a function of attentional load (i.e., number of tracked objects) and days of measurement. **(C)** Pupil size increases relative to a passive viewing condition (i.e., tracking no targets) as a function of attentional load and days of measurement. Error bars in **(B,C)** are standard error of the mean. All figures have been adapted from Wahn et al. (2016a).

Limitations of visuospatial attention have been investigated in a wide variety of visuospatial tasks. One task that has been suggested to be highly suitable [among others such as response-competition tasks (Lavie, 2005, 2010; Matusz et al., 2015), or orthogonal cueing tasks (Spence and Driver, 2004; Spence, 2010)] to investigate visuospatial attentional processing is the “Multiple Object Tracking” (MOT) task (Pylyshyn and Storm, 1988; Yantis, 1992) (see Figure 1A, for a typical trial logic) as the attentional load can be systematically varied (i.e., by varying the number of targets that need to be tracked) while keeping the perceptual load constant (i.e., the total number of displayed objects) (Cavanagh and Alvarez, 2005; Arrighi et al., 2011; Wahn and König, 2015a,b). Notably, apart from spatial attentional demands, the MOT task also involves anticipatory processes (i.e., predicting the trajectories of the targets' movements) (Keane and Pylyshyn, 2006; Atsma et al., 2012). However, as in several studies investigating the MOT task the trajectories of targets also do change randomly (e.g., in Wahn and König, 2015a,b), the MOT task at least in these cases primarily involves spatial attentional processing. The general finding across studies is that with an increasing number of targets, performance in the MOT task systematically decreases (see Figure 1B), suggesting a limit of visuospatial attentional resources (Alvarez and Franconeri, 2007; Wahn et al., 2016a). Moreover, these capacity limitations are stable across several repetitions of the experiment on consecutive days (Wahn et al., 2016a, see Figure 1B) and over considerably longer periods of time (Alnæs et al., 2014).

The behavioral findings from the MOT task have been corroborated by studies looking at the physiological correlates of attentional processing. A prominent physiological correlate of attentional processing are pupil sizes (Heinrich, 1896; Kahneman and Beatty, 1966; Beatty, 1982; Hoeks and Levelt, 1993; Wierda et al., 2012; Mathôt et al., 2013; Alnæs et al., 2014; Lisi et al., 2015; Mathôt et al., 2016). Increases in pupil sizes have been shown to be associated with increases in attentional load in recent studies that used the MOT task (Alnæs et al., 2014; Wahn et al., 2016a). Specifically, it has been shown that when participants perform the MOT task at varying levels of attentional load, pupil sizes systematically increase with attentional load and these increases are consistently found for measurements on consecutive days (Wahn et al., 2016a, see Figure 1C). Apart from these studies investigating changes in pupil size, researchers also investigated physiological correlates of attentional processing using fMRI and EEG. Researchers found that parietal regions in the brain typically associated with attentional processing were active when participants performed the MOT task (Jovicich et al., 2001; Howe et al., 2009; Jahn et al., 2012; Alnæs et al., 2014) but notably also for several other spatial tasks (Mishkin and Ungerleider, 1982; Livingstone and Hubel, 1988; Maeder et al., 2001; Reed et al., 2005; Ahveninen and et al., 2006; Ungerleider and Pessoa, 2008), suggesting that performing the MOT task requires processing of brain regions typically associated with visuospatial attention. Moreover, several EEG studies have identified neural correlates whose activity rises with increasing attentional load in the MOT task (Sternshein et al., 2011; Drew et al., 2013).

In sum, the MOT task has served to assess visuospatial limitations of attentional resources in a number of studies (Alvarez and Franconeri, 2007; Alnæs et al., 2014; Wahn et al., 2016a) and their physiological correlates (Jovicich et al., 2001; Howe et al., 2009; Jahn et al., 2012; Alnæs et al., 2014; Wahn et al., 2016a). In the following, we discuss how the use of the MOT and other spatial tasks has been extended to investigate spatial attentional resources across multiple sensory modalities.

## 3. Circumventing limitations of visuospatial attention

A question that has been extensively investigated in multisensory research is whether there are distinct pools of attentional resources for each sensory modality or one shared pool of attentional resources for all sensory modalities. Studies have found empirical support for the hypothesis that there are distinct resources (Duncan et al., 1997; Potter et al., 1998; Soto-Faraco and Spence, 2002; Larsen et al., 2003; Alais et al., 2006; Hein et al., 2006; Sinnett et al., 2006; Talsma et al., 2006; Van der Burg et al., 2007; Keitel et al., 2013; Finoia et al., 2015) as well as for the hypothesis that there are shared resources (Jolicoeur, 1999; Arnell and Larson, 2002; Soto-Faraco et al., 2002; Arnell and Jenkins, 2004; Macdonald and Lavie, 2011; Raveh and Lavie, 2015). In principle, if there are separate pools of attentional resources, attentional limitations in one sensory modality can be circumvented by distributing attentional processing across several sensory modalities. Conversely, if there is only one shared pool of attentional resources for all sensory modalities, attentional limitations in one sensory modality cannot be circumvented by distributing attentional processing across several sensory modalities.

The question of whether there are shared or distinct attentional resources across the sensory modalities has often been investigated using dual task designs (Pashler, 1994). In a dual task design, participants perform two tasks separately (“single task condition”) or at the same time (“dual task condition”). The extent to which attentional resources are shared for two tasks is assessed by comparing performance in the single task condition with performance in the dual task condition. If the attentional resources required for the two tasks are shared, task performance should decrease in the dual task condition relative to the single task condition. If attentional resources required for the two tasks are distinct, performance in the single and dual task conditions should not differ. In multisensory research, the two tasks in a dual task design are performed either in the same sensory modality or in different sensory modalities. The rationale of the design is that two tasks performed in the same sensory modality should always share attentional resources while two tasks performed in separate sensory modalities may or may not rely on shared attentional resources. That is, if attentional resources are distinct across sensory modalities, tasks performed in two separate sensory modalities should interfere less than tasks performed in the same sensory modality.

**Figure 2:**
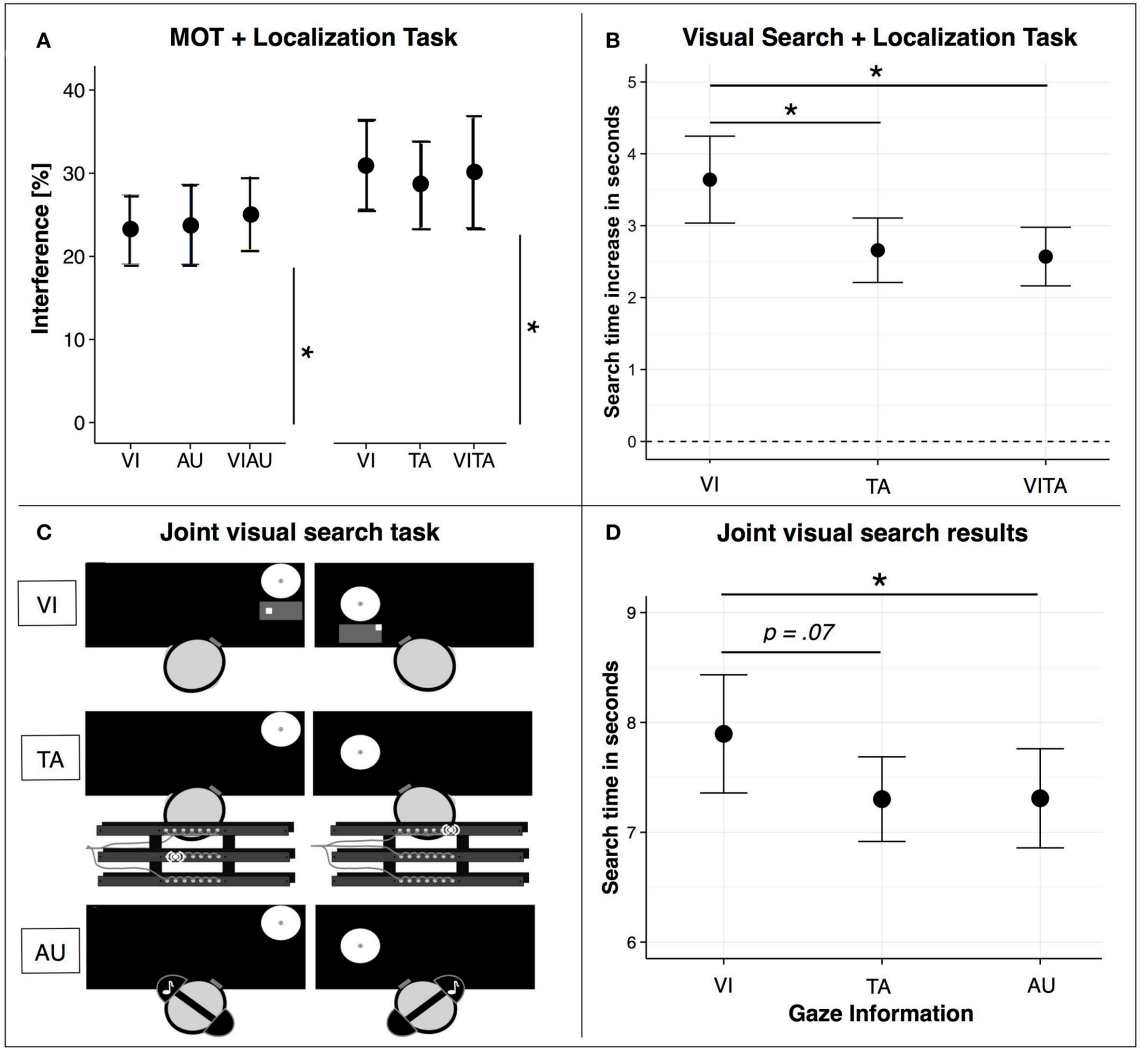
**(A)** Dual task interference when participants perform the MOT task either in combination with a visual (VI), tactile (TA), audiovisual (VIAU), or visuotactile (VITA) localization task. Interference is measured as the reduction in performance between single and dual task conditions. In particular, the reduction in performance for both tasks (i.e., MOT and localization task) are combined by taking the Euclidean distance between the performances in the single and dual task conditions, separately for each combination of tasks (MOT+VI, MOT+AU, MOT+TA, MOT+VIAU, MOT+VITA). **(B)** Search time increase relative to performing the visual search task alone when participants perform the same task either in combination with the VI, TA, or VITA localization task. **(C)** Joint visual search task conditions. Co-actors jointly searched for a target among distractors on two separate computer screens. A black mask was applied to the whole screen and only the currently viewed location was visible to the co-actors. Co-actors received the information about where their co-actor was looking either via a visual map (VI) that was displayed below their viewed location, via vibrations on a vibrotactile belt (TA), or via tones received through headphones (AU). **(D)** Joint visual search results. Search performance (i.e., time of the co-actor who found the target first) as a function of the sensory modality (VI, TA, or AU) in which the gaze information was received. Error bars in **(A,B,D)** are standard error of the mean. ^*^Indicate significant comparisons with an alpha of .05. **(A)** has been adapted from Wahn and König (2015a,b), **(B)** from Wahn and König (2016), and **(C,D)** from Wahn et al. (2016c).

In the following, we will focus on research that has investigated how limitations in attentional resources for visuospatial attention can be circumvented by distributing information processing across sensory modalities using dual task designs. Several researchers suggested that a factor that influences the allocation of attentional resources across sensory modalities is the task-specific type of attentional processing (Bonnel and Hafter, 1998; Chan and Newell, 2008; Arrighi et al., 2011; Wahn and König, 2016; Wahn et al., 2017c). That is, the allocation of attentional resources depends on whether tasks performed in separate sensory modalities require object-based attention or spatial attention (for a recent review, see Wahn and König, 2017). In recent studies (Arrighi et al., 2011; Wahn and König, 2015a,b), this task-dependency in attentional resource allocation has been tested in a dual task design involving a visuospatial task (i.e., a MOT task). In particular, the MOT task was performed either alone or in combination with a secondary task that was either performed in the visual, auditory, or tactile sensory modalities. The secondary task either required object-based attention (i.e., the secondary task was a discrimination task) or spatial attention (i.e., the secondary task was a localization task). When participants performed the MOT task in combination with an object-based attention task in another sensory modality (i.e., an auditory pitch discrimination task), distinct attentional resources were found for the visual and auditory modalities (Arrighi et al., 2011). However, in studies in which participants performed the MOT task in combination with either a tactile (Wahn and König, 2015b) or auditory localization task (Wahn and König, 2015a), findings suggest that attentional resources are shared across the visual, tactile, and auditory sensory modalities. In particular, results showed that regardless of whether two spatial attention tasks were performed in two separate sensory modalities or the same sensory modality, tasks equally interfered with each other (see Figure 2A).

Further support for these conclusions was provided in another study (Wahn and König, 2016). In contrast to earlier studies (Wahn and König, 2015a,b), this time an object-based attention task was combined with a spatial attention task. In particular, participants performed a visual search task either in combination with a visual or tactile localization task. In line with the findings above (Arrighi et al., 2011), participants performed the visual search task faster in combination with the tactile localization task than in combination with the visual localization task (see Figure 2B). These findings suggest that attentional resources for the sensory modalities are distinct when tasks involve different types of attentional processing, i.e. object-based and spatial attentional processing.

In sum, the findings discussed above suggest that the allocation of attentional resources across sensory modalities (i.e., whether they are shared or distinct) depends on what type of attentional processing is required in a task. In particular, if tasks only require spatial attentional processing, findings suggest that attentional resources are shared across sensory modalities (Wahn and König, 2015a,b). However, if tasks also require object-based attentional processing, findings suggest that attentional resources are distinct across the sensory modalities (Arrighi et al., 2011; Wahn and König, 2016). Importantly, limitations in visuospatial attention can be circumvented by distributing attentional processing across sensory modalities if tasks involve object-based as well as spatial attentional processing.

Apart from the task-dependency, we also want to emphasize that there are several other factors that influence attentional processing such as motor demands (Marois and Ivanoff, 2005; Dux et al., 2006) and the sensory modality in which task load is increased (Rees et al., 2001; Macdonald and Lavie, 2011; Molloy et al., 2015; Raveh and Lavie, 2015) (for a detailed discussion, see Wahn and König, 2017). Another important factor to consider is the age of participants. Findings of a recent study (Matusz et al., 2015) suggested that conclusions about the distribution of attentional resources across the sensory modalities for adults do not necessarily generalize to children. In addition, we want to note that another effective means to circumvent limitations in one sensory modality is by providing redundant information via several sensory modalities, thereby taking advantage of the behavioral benefits of multisensory integration (i.e., faster reaction times and a higher accuracy) (Meredith and Stein, 1983; Ernst and Banks, 2002; Helbig and Ernst, 2008; Stein and Stanford, 2008; Gibney et al., 2017). The process of multisensory integration has been argued to be independent of top-down influences (Matusz and Eimer, 2011; De Meo et al., 2015; ten Oever et al., 2016) and be robust against additional attentional demands (Wahn and König, 2015a,b) for low-level stimuli (for more general reviews on the topic, see van Atteveldt et al., 2014; Chen and Spence, 2016; Macaluso et al., 2016; Tang et al., 2016), making it highly suitable to circumvent limitations within one sensory modality.

## 4. Circumventing limitations of visuospatial attention in joint tasks

In previous sections, we have reviewed studies in which participants perform a task alone. However, in many situations in daily life, tasks are performed jointly by two or more humans with a shared goal (Sebanz et al., 2006; Vesper et al., 2017). For instance, when two humans carry a table together (Sebanz et al., 2006), search for a friend in a crowd (Brennan et al., 2008), or play team sports such as basketball or soccer. In such joint tasks, humans often achieve a higher performance than the better individual would achieve alone (i.e., a collective benefit) (Bahrami et al., 2010). Collective benefits have been investigated in several task domains such as visuomotor tasks (Knoblich and Jordan, 2003; Masumoto and Inui, 2013; Ganesh et al., 2014; Skewes et al., 2015; Rigoli et al., 2015; Wahn et al., 2016b), decision-making tasks (Bahrami et al., 2010, 2012a,b), and visuospatial tasks (Brennan et al., 2008; Neider et al., 2010; Brennan and Enns, 2015; Wahn et al., 2016c, 2017b).

Regarding visuospatial tasks, several studies have investigated joint performance in visual search tasks (Brennan et al., 2008; Neider et al., 2010; Brennan and Enns, 2015; Wahn et al., 2016c). In particular, Brennan et al. (2008) investigated how performance in a joint visual search task depends on how information is exchanged between two co-actors. In a joint visual search task, two co-actors jointly search for a target stimulus among distractor stimuli. Brennan et al. (2008) found that co-actors performed the joint search the fastest and divided the task demands most effectively in the condition where they received gaze information (i.e., a continuous display of the co-actor's gaze location), suggesting that co-actors highly benefit from receiving spatial information about the actions of their co-actor (also see Wahn et al., 2017b).

The task demands in the joint visual search task as employed by Brennan et al. (2008) involve a combination of object-based attention (i.e., discriminate targets from distractors in the visual search task) and spatial attention (i.e., localize where the co-actor is looking using the gaze information). As reported above, findings in multisensory research suggest that limitations of visuospatial attention can be effectively circumvented by distributing information processing across sensory modalities if processing involves a combination of object-based attention and spatial attention (Arrighi et al., 2011; Wahn and König, 2016). In a recent study (Wahn et al., 2016c), these findings from multisensory research were applied to a joint visual search task setting similar to the one used by Brennan et al. (2008). In particular, researchers investigated whether joint visual search performance is faster when actors receive information about their co-actor's viewed location via the auditory or tactile sensory modality compared to when they receive this information via the visual modality (see Figure 2C). Researchers found that co-actors searched faster when they received the viewing information via the tactile or auditory sensory modalities than via the visual sensory modality (see Figure 2D). These results suggest that findings from multisensory research mentioned above (Arrighi et al., 2011; Wahn and König, 2016) can be successfully applied to a joint visuospatial task.

## 5. Conclusions and future directions

The aim of the present review was to review recent studies investigating limitations in visuospatial attention. These studies have reliably found limitations of visuospatial attention and physiological correlates whose activity rises with increasing visuospatial attentional demands (Sternshein et al., 2011; Drew et al., 2013; Alnæs et al., 2014; Wahn et al., 2016a). Findings from multisensory research have demonstrated that such limitations of visuospatial attention can be circumvented by distributing information processing across sensory modalities (Arrighi et al., 2011; Wahn and König, 2015a,b, 2016) and these findings are applicable to joint tasks (Wahn et al., 2016c).

Apart from the study above (Wahn et al., 2016c), other studies on joint action have investigated how the use of multisensory stimuli (e.g., visual and auditory) can serve to facilitate joint performance (Knoblich and Jordan, 2003) and how the process of multisensory integration is affected by social settings (Heed et al., 2010; Wahn et al., 2017a). However, these studies have not investigated how distributing information processing across sensory modalities potentially could facilitate joint performance. We suggest that future studies could further investigate to what extent findings from multisensory research are applicable to joint tasks. In particular, attentional limitations may be circumvented in every joint task that involves a combination of object-based and spatial attentional processing in the visual sensory modality, thereby possibly facilitating joint performance.

The possibility to circumvent limitations of visuospatial attention is also relevant for many real-world tasks that require visuospatial attention such as car-driving (Spence and Read, 2003; Kunar et al., 2008; Spence and Ho, 2012), air-traffic control (Giraudet et al., 2014), aviation (Nikolic et al., 1998; Sklar and Sarter, 1999), navigation (Nagel et al., 2005; Kaspar et al., 2014; König et al., 2016), or rehabilitation (Johansson, 2012; Maidenbaum et al., 2014). Notably, for applying findings to real-world tasks additional factors such as how much the task was practiced (Ruthruff et al., 2001; Chirimuuta et al., 2007) or memorized (Matusz et al., 2017) should be taken into account as real-world tasks are often highly practiced and memorized. More generally, in such scenarios limitations of visuospatial attention could be effectively circumvented by distributing attentional processing across sensory modalities, thereby improving human performance and reducing the risk of accidents.

## Author contributions

Drafted the manuscript: BW. Revised the manuscript: BW and PK.

### Conflict of interest statement

The authors declare that the research was conducted in the absence of any commercial or financial relationships that could be construed as a potential conflict of interest.
